# Can Epigenetics Predict Drug Efficiency in Mental Disorders?

**DOI:** 10.3390/cells12081173

**Published:** 2023-04-17

**Authors:** Gil Ben David, Yam Amir, Randa Salalha, Lital Sharvit, Gal Richter-Levin, Gil Atzmon

**Affiliations:** 1 Sagol Department of Neurobiology, Faculty of Natural Sciences, University of Haifa, 199 Aba Khoushy Ave., Mount Carmel, Haifa 3498838, Israel; gili.bd@gmail.com (G.B.D.); randa.sa@gmail.com (R.S.);; 2 Department of Human Biology, Faculty of Natural Sciences, University of Haifa, 199 Aba Khoushy Ave., Mount Carmel, Haifa 3498838, Israel; yamamir1138@gmail.com (Y.A.);; 3 Department of Psychology, Faculty of Social Sciences, University of Haifa, 199 Aba Khoushy Ave., Mount Carmel, Haifa 3498838, Israel; 4 Integrated Brain and Behavior Research Center (IBBR), University of Haifa, 199 Aba Khoushy Ave., Mount Carmel, Haifa 3498838, Israel

**Keywords:** psychiatric disorders, personalized medicine, epigenetics

## Abstract

Psychiatric disorders affect millions of individuals and their families worldwide, and the costs to society are substantial and are expected to rise due to a lack of effective treatments. Personalized medicine—customized treatment tailored to the individual—offers a solution. Although most mental diseases are influenced by genetic and environmental factors, finding genetic biomarkers that predict treatment efficacy has been challenging. This review highlights the potential of epigenetics as a tool for predicting treatment efficacy and personalizing medicine for psychiatric disorders. We examine previous studies that have attempted to predict treatment efficacy through epigenetics, provide an experimental model, and note the potential challenges at each stage. While the field is still in its infancy, epigenetics holds promise as a predictive tool by examining individual patients’ epigenetic profiles in conjunction with other indicators. However, further research is needed, including additional studies, replication, validation, and application beyond clinical settings.

## 1. Introduction

### 1.1. Personalized Medicine

Personalized medicine is a rapidly expanding medical discipline that is guided by each individual’s unique clinical, genetic, genomic, and environmental profile. The primary goal of personalized medicine is to improve a person’s medical care and outcomes by tailoring care to a specific patient. It is based on the idea that each person has unique molecular, physiological, environmental, and behavioral characteristics [1]. However, predicting the optimal treatment for any patient with any disease remains challenging.

Technological advances have brought us closer to practicing personalized medicine over the last two decades. For example, genome-wide association studies (GWASs) have revived pharmacogenetics, a term used for the first time in the 1950s [2], which is now one of the pillars of personalized medicine.

GWASs have transformed the approach to personalized medicine by moving away from studying a limited number of potential genes (the candidate gene approach) to a comprehensive search for hundreds of thousands of genetic markers (unbiased genomic screening) [3]. This new approach has led to practical applications in the clinic and significant advancements, such as the utilization of Cytochrome P450 genotypes to predict an individual’s drug metabolism phenotype. In addition, through GWAS analysis, numerous pharmacogenomic biomarkers, particularly those used in cancer treatment, have been identified [4]. However, despite the significant advancements made through GWASs, it is important to note that, counterintuitively, genetic variation alone explains only a limited proportion, approximately 20–30%, of inter-individual differences in drug response [5]. This is due to the complexity of living organisms and the limitations of genotype information. As described by Kronfol et al., two broad complexities that are not addressed by genotype information are: (1) spatial variation or cell-specific variation: the diversity of cell types and functions within the same organism. For example, humans are composed of multiple cell types, each with its own unique function, which leads to diversity in phenotypic expression; and (2) temporal or developmental variation: the changes in biological function or phenotypic expression throughout the organism’s lifetime. For example, humans take on different forms during early and later life stages, yet the same genome is present in all nucleated cells at all time points [6]. Therefore, while genetic variation is an essential aspect of drug response, it is not the only factor to consider; a person’s response to a drug is a complex process that almost certainly results from the interaction of genetic and environmental factors [7]. Pharmacogenetics, which examines the role of genetic variation in drug response, can provide insight into one variable in the equation of drug response. Epigenetics, which explores how environmental factors influence gene regulation and expression, can offer insight into another variable in the equation. The field of pharmacoepigenetics, a relatively new area of drug discovery, combines the study of both pharmacogenetics and epigenetics to better understand the multiple variables that contribute to drug response [5].

### 1.2. Epigenetics

The term “epigenetics” refers to DNA alterations (which can be triggered by environmental signals) that affect gene expression without changing the DNA linear sequence. Among these changes are histone modifications, non-coding RNA, hydroxymethylation, and, most notably, methylation. DNA methylation is a covalent DNA modification, catalyzed by the DNA methyltransferase (DNMT, DNA MTase) enzyme family [8], that places a methyl group on cytosine nucleotides at the c5 position [8] and is most prominent among mammals [5]. Epigenetic research has become an important component of modern medicine due to its emphasis on examining the relationship between genetic and environmental risk factors [9]. Methylation of genomic DNA has primarily been observed in the context of cytosine methylation in CpG sites and is regarded as the most common epigenetic modification [10]. CpG sites are loci in DNA sequences where a cytosine nucleotide occurs first, followed by a guanine nucleotide. DNA methylation, a persistent epigenome component, maintains gene expression levels to establish and stabilize cell-specific phenotypes [11]. A methylation site can influence gene expression in various ways [12,13,14]. However, most research focuses on methylated promoters which are typically associated with transcriptional suppression [15,16].

In addition to DNA methylation, another important epigenetic mechanism is the regulation of gene expression by non-coding RNAs (ncRNAs). Non-coding RNAs are RNA molecules that do not encode proteins, and they can act as post-transcriptional regulators of gene expression. Among the various types of ncRNAs, MicroRNA (miRNA) is the most well-studied. Most miRNAs are produced by the transcription of DNA sequences into primary miRNAs, which are then transformed into precursor miRNAs and mature miRNAs [17]. Mature miRNAs bind to the 3’ untranslated region (3’UTR) of target mRNAs, silencing or suppressing gene expression. miRNA expression varies among tissues and may be influenced by inflammation, disease, and medical substances. MiRNAs control 30–60% of all human protein-coding genes and have therapeutic potential regarding pharmacological efficacy and safety [18].

This review will delve into the intricacies of DNA methylation and miRNA as the most extensively studied mechanisms of epigenetic modulation.

### 1.3. The Importance of Personalized Medicine in Psychiatric Disorders

While the majority of research utilizing epigenetics in personalized medicine has focused on cancer [5], it has the potential to be a valuable approach in developing individualized treatments for psychiatric disorders as well. First, the effects of psychiatric diseases, which affect many people worldwide, impose a significant burden on patients, their families, and society. According to epidemiological studies, anxiety disorders, including phobias, panic disorders (PD), post-traumatic stress disorder (PTSD), and obsessive–compulsive disorder (OCD), affect nearly 22.2% of the people in the United States. Mood disorders, such as major depressive disorders (MDD) and bipolar disorder, affect an estimated 9.4% of people [19]. The estimated global direct and indirect economic costs of mental illnesses in 2010 were USD 2.5 trillion. Mental disorders account for more economic costs than chronic somatic diseases, such as cancer or diabetes, and their costs are expected to increase exponentially over the next 10 years [20].

Second, the selection of an appropriate treatment for a patient is often based on a trial-and-error approach. It is not uncommon for patients to experience a delay in the relief of symptoms. For instance, when administering a selective serotonin reuptake inhibitor (SSRI) antidepressant, it is generally accepted that it takes at least four weeks to observe a response and an average of six weeks to achieve remission. However, there have been instances where it took longer than 12 weeks for patients to experience the desired therapeutic effect [21]. Further, approximately two-thirds of patients do not respond well to the initial round of treatment, and 20–30% do not respond at all, despite varying doses and repeated treatments [22]. Aside from the challenges mentioned above, there are pharmacological side effects, such as weight gain, fatigue, drowsiness, akathisia, and sexual dysfunction [23]. As it takes a while for a patient to experience the effect of a given medication, along with the lack of confidence that the specific drug will indeed have an effect and the unpleasant side effects, many patients simply discontinue treatment [23,24,25]. Thus, it is necessary to discover biomarkers to guide treatment selection and detect potential poor responders in advance.

Concurrent with the limited success of pharmacogenetic studies in predicting drug efficacy in mental disorders [26,27], a few studies have attempted to predict the efficacy of treatments using epigenetic profiles (Tables S1–S3).

## 2. Methods

In this study, we first gathered articles from the PubMed database using a comprehensive search query focusing on mental disorders, epigenomics, and precision medicine. The search query aimed to identify relevant literature on the efficacy and outcomes of various treatment options, clinical trials, and personalized medicine approaches in the context of epigenetics.

**Scheme 1:**
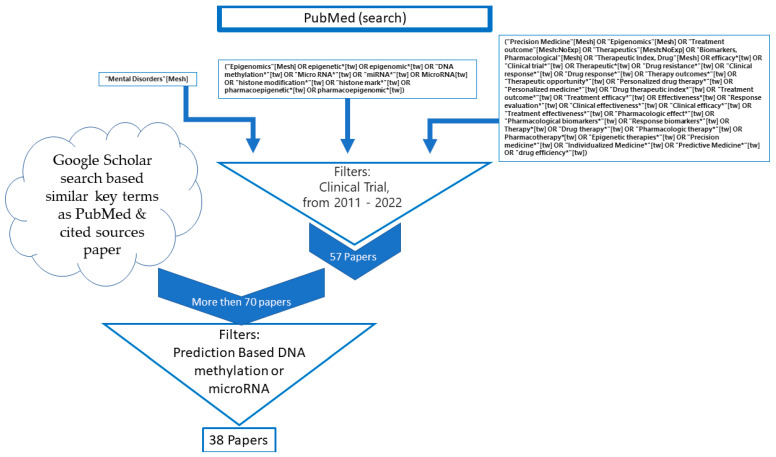
Schematic diagram of literature review.

We applied filters to our search, limiting the results to clinical trials conducted between 2011 and 2022, sorted by the most recent publications. This initial search yielded 57 papers. We then sought additional articles from the cited sources within these papers and by using Google Scholar, employing search terms related to specific mental disorders, epigenetics, and drug efficacy (Scheme 1).

Although we did not carry out a full systematic collection, we examined a wide range of articles. We selected articles for inclusion based on certain criteria, such as the focus on human subjects and the ability to predict drug response using dichotomous or continuous parameters of symptoms informed by DNA methylation or microRNA data. We reviewed over 70 articles, ultimately including 38 in our analysis.

**Figure 1:**
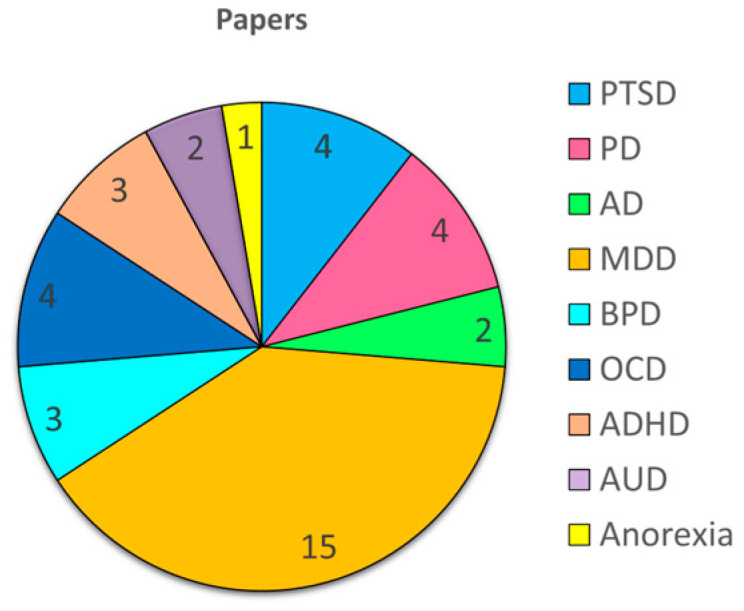
Distribution of reviewed papers by psychiatric disorder: a pie chart illustrating the number of articles included in this review that discuss each specific mental health condition.

The majority of the reviewed literature focused on depression (18 articles); followed by anxiety-related disorders, such as PTSD, AD, and PD (10 articles); OCD (4 articles); border-line personality disorder (3 articles); ADHD (3 articles); alcohol use disorder (2 articles); and anorexia (1 article) (Figure 1).

We organized the papers based on subject matter, starting with early attempts to apply epigenetics in determining treatment efficacy and then progressing to the findings and the frequency with which specific topics were addressed in the articles. The selected papers were used as references for the topics discussed in the article.

## 3. Epigenetics Prediction of Treatment Efficiency

### 3.1. Anxiety Disorders, Panic Disorders, Phobias, and PTSD

An early attempt to predict treatment efficiency using epigenetics was conducted by Yehuda et al. (2013). Building on previous research that had identified various aspects of the glucocorticoid receptor (GR) gene (*NR3C1*) and *FKBP5* genes as being implicated in post-traumatic stress disorder (PTSD), Yehuda et al. investigated whether it was possible to predict the success of 12 weeks of prolonged exposure (PE) psychotherapy among combat veterans diagnosed with PTSD using DNA methylation mapping of *NR3C1* and *FKBP5* genes. The study found that methylation of the GR gene (*NR3C1*) exon 1F promoter, assessed at pre-treatment, predicted treatment outcome, such that responders had higher methylation at baseline compared to non-responders. In contrast, methylation of the *FKBP5* gene (*FKBP51*) exon 1 promoter region showed a decrease in association with recovery but did not predict treatment response [28]. These findings were partially repeated in a study by Pape et al., who studied female PTSD patients before and after five months of treatment with a CRF1 receptor antagonist. The study found that higher baseline methylation of *NR3C1* was associated with better response to the antagonist, but this association was only observed in participants who had also been abused as children. In contrast to the findings of Yehuda et al., Pape et al. did not find any association between baseline or change in methylation levels of *FKBP5* and treatment outcome or symptom improvement [29]. However, after 9 weeks of mindfulness-based stress reduction therapy, *FKBP5* intron 7 methylation decreased in PE-responsive PTSD patients, while methylation increased in non-responders. Moreover, improvements in PTSD symptoms from pre- to post-therapy were negatively linked with changes in methylation at the same location [30]. In a study by Yang et al., partially supporting results were found, showing that decreased methylation in the *FKBP5* gene body (two CpG sites near exons) was associated with a decrease in PTSD symptoms in responders, but no change was observed in non-responders to psychotherapy with or without cortisol augmentation. Additionally, this study found that, unlike previous findings, the methylation levels of *NR3C1* at pre-treatment were marginally significantly lower in responders than in non-responders and were unrelated to recovery [31].

Additional findings for predicting treatment efficacy using DNA methylations highlighted the *PEX5* and *ALOX15B* genes. These genes were found to have decreased methylation levels in individuals who responded positively to psychotherapy treatment, as evidenced by changes in PTSD symptom severity from pre- to post-treatment. Additionally, the gene *SDK1* was found to differentiate responders and non-responders both before and after treatment, with methylation levels increasing significantly in non-responders and decreasing in responders [31]. In addition, another study by Pape et al. (2018) suggested that baseline methylation of the *CRHR1* gene may only serve as a marker of symptom changes, as it did not predict treatment outcome with a CRF1 receptor antagonist among female PTSD patients [29].

Overall, the main findings of the studies mentioned above concern *NR3C1* and *FKBP5*. *FKBP5* is essential for stress reactivity via negative regulation of the hypothalamus–pituitary–adrenal (HPA) axis [32,33], and dysregulation of this process has been widely linked to stress-related disorders, such as anxiety disorders and phobias. Thus, it is not surprising that further research has revealed the potential efficacy of treatment based on the epigenetic regulation of *FKBP5* in other disorders. For example, among patients undergoing exposure-based CBT for phobia with or without panic disorder, those with a lower percentage of DNA methylation at the CpG site 5 of intron 7 had a better response to therapy (lower symptom intensity), whereas those with higher DNA methylation over time had a worse treatment outcome [34]. Similar results were observed in children with anxiety disorders (ADs) following CBT treatment [35]. These findings are in-line with other works [28,29,31], although these are different diagnoses.

However, the findings regarding the relationship between symptom improvement and methylation in *FKBP5* indicate a consistent pattern regardless of the location of methylation within the gene (intron or exon), deviating from the general trend [12,13,14]. Furthermore Roberts et al. did not find an association between DNA methylation levels of *FKBP5* and gene expression [34].

Another study by the same research group investigating anxiety disorder and the prediction of treatment efficiency found differences in *SERT* methylation change in association with response to CBT. Specifically, the study found that a CpG site upstream of the *SERT* promoter showed increased methylation over the course of CBT treatment among responders, while non-responders showed a decrease in DNA methylation [36].

Many of the studies reviewed here have been focused on trying to predict the outcome of CBT. CBT aims to increase emotional control and build personal coping mechanisms for handling present difficulties by confronting and modifying cognitive distortions (such as beliefs, ideas, and attitudes) [37]. This technique is a widely used therapy method for many mental health conditions, including anxiety disorder and PD. Two different experimental studies, by the same research group, aimed to predict CBT efficiency for PD patients using DNA methylations [38,39]. In the first study, conducted in 2016, it was found that baseline methylation levels differed significantly between therapy responders and non-responders at two CpG sites of the *MAOA* gene. However, the study did not specify the direction of the difference or the location of the CpG sites (exon 1 or intron 1). Additionally, the study found that PD severity was negatively correlated with *MAOA* methylation at baseline, and non-response was associated with a significant drop in *MAOA* methylation, while response was associated with a further rise in *MAOA* methylation throughout treatment, up to the level of healthy controls. This finding was replicated by an independent sample, which showed an inverse correlation between an increase in *MAOA* methylation and a decrease in agoraphobic symptoms throughout the treatment [39]. The second study, conducted in 2019, analyzed the effects of six-week CBT on DNA methylation alterations and did not demonstrate a significant epigenome-wide impact. However, evidence suggests that methylation of a CpG site located in intron 1 of the *IL1R1* gene increased following CBT in individuals who responded well to therapy, but this was not observed in non-responders [38]. It should be noted that it is likely that the results of the second study could not be properly compared to the earlier study because the two investigations used different techniques for analyzing methylation. A study by Min et al. (2019) investigated the association between epigenetic changes and treatment outcomes in PD patients. Unlike most of the studies reviewed to this point, which all focused on methylation, this study specifically looked at miRNA, and the treatment used was pharmacological. The study found that changes in miR-25-3p expression were positively related to the improvement of patients’ expected anxiety and impairment of social anxiety. Additionally, changes in miR-660-5p were positively associated with improvement of patient memory and attention and impairment of social anxiety. The study also found several more findings regarding miR-451a, miR-144-5p, miR-1, and miR-148b-5p, which were associated with the effect on patients with PD as a result of sertraline treatment [40]. It is important to note that in this study all the subjects responded at one level or another, whether total remission or another improvement; thus, no direct comparison was made between responders and non-responders. It is worth mentioning that Pape et al. (2018) and Yang et al. (2021) also studied pharmacological treatment (cortisol augmentation) but found no effect.

SSRIs are widely used antidepressants that are commonly prescribed to treat various mental health conditions, such as generalized anxiety disorder (GAD), PD, and severe phobias. It is important to note that even though SSRIs are the first-line pharmacological treatment for PTSD and the only type of drug currently approved by the Food and Drug Administration (FDA) for PTSD [41,42], there have been no studies attempting to predict the efficacy of different SSRI types in the treatment of PTSD. Reviewed papers are summarize in Table S1.

Most research on predicting treatment effectiveness using epigenetics has been carried out in the field of depression. In the next section, we will review some of these findings.

### 3.2. Major Depressive Disorder (MDD)

In 2013 Powell et al., performed research involving 12 weeks of escitalopram or nortriptyline treatment among 113 MDD patients. The study revealed that lower levels of DNA methylation at a specific CpG site of *ILI11* were related to a better response to antidepressants. In addition, higher levels of DNA methylation at another CpG site of the same gene were associated with a better response to escitalopram but a poorer response to nortriptyline [43]. To our knowledge, this study is the only one that has identified a specific methylation site that distinguishes between individuals who respond positively to one drug versus another.

*SLC6A4* is one of the key genes in the field of MDD, and while there are some contradictory results, most of the studies corroborate one another [44,45,46,47]. Kang et al. found no more than a slight trend toward higher methylation levels among individuals who showed minor improvement in MDD symptoms after 12 weeks of antidepressant treatment [44]. This finding is supported by another study that showed that at the same genomic region, one CpG site had higher methylation among MDD patients who had improved symptoms after 6 weeks of antidepressant treatment compared to those who had a poorer response. Additionally, there was a positive correlation between pre-treatment methylation at the same CpG site and the change in symptoms [45]. This research included a total of 40 participants with MDD. Domschke et al. further supported these findings by testing methylation of nine CpG sites in the *SLC6A4* gene region from pre-treatment blood samples of 94 patients treated with escitalopram and other drugs for 6 weeks and were able to distinguish treatment responders from non-responders [47]. These results were almost fully replicated in 2021 among 236 MDD patients treated with SSRI/SNRI [46]. While most findings addressed *SLC6A4*, another huge player in the field of psychiatric disorders in general and specifically in depression is *BDNF* [48]. Two studies have investigated the potential of using *BDNF* methylation status to predict treatment response in MDD patients. The first study by Tadić et al. (2014) found that non-responders to a 6-week treatment with antidepressants had significantly reduced CpG methylation in exon 4 of *BDNF* compared to responders at baseline, but there were no differences in methylation before and after treatment [49]. The second study by Wang et al. (2018) also found that lower methylation at the promoter of *BDNF* was associated with poorer response to escitalopram. However, in contrast to that of exon 4 of *BDNF* mentioned in the former study, *BDNF* promoter methylation status was found to be significantly lower at baseline compared to after 8 weeks of treatment [50].

While the previously mentioned studies focused on specific genes, a study conducted by Ju et al. aimed to investigate potential epigenetic biomarkers of the effects of SSRI antidepressant medications in patients with MDD using genome-wide DNA methylation analysis. The study employed the Infinium MethylationEPIC Beadchip, and the trial lasted for eight weeks of escitalopram treatment. The research team identified three differentially methylated positions (DMPs) in the *CHN2* and *JAK2* genes most significantly associated with changes in mRNA expression. The findings were then validated using a targeted sequencing approach and partially replicated in an external cohort undergoing similar trails but with a combination of both SSRIs and SNRIs. Notably, the *CHN2* gene was the only one that came up in both the first and second cohorts, indicating that it may play a crucial role in MDD and SSRI treatment. Notably, the *CHN2* gene has not been identified in previous therapy prediction studies. However, it is important to note that the study has some limitations that should be considered. For example, the study utilized stringent differential methylation and expression cut-offs, which could have influenced the results. Additionally, there were differences between the two cohorts, including the fact that those in the second cohort who were treated with an SNRI drug were not included in the first cohort, and the methods of diagnosing the symptoms of depression were not identical, which may have led to a difference in the method of separating responders from non-responders. Another limitation is that the technical methods used for differential methylation analyses do not distinguish between hydroxymethylated cytosines and methylated cytosines, which could impact the accuracy of the results [51].

It is worth noting that some studies have failed to find predictive ability in certain genes when it comes to antidepressant treatment. For example, a study by Domschke et al. (2015) found that methylation at the baseline of the promoter, exon1, and intron 1 of the *MAOA* gene did not predict or associate with a 6-week escitalopram treatment [52]. Similarly, Kahl et al. (2016) found that methylation of *SLC2A1* and *SLC2A4* (also known as *GLUT1* and *GLUT4*) did not predict response to 6 weeks of CBT as a treatment for depression [53].

In addition to evidence for DNA methylation in the treatment of MDD, studies have also found changes in the expression of various miRNAs in MDD patients. One study found that MDD patients who responded to 8 weeks of escitalopram treatment had different levels of miR-1202 at baseline compared to those who did not respond to treatment. Specifically, the patients who responded to treatment (remitters) had lower levels of miR-1202 at baseline compared to non-responders. Additionally, after 8 weeks of therapy, remitters had increased levels of miR-1202 expression, while non-responders did not show any changes. Furthermore, changes in depression intensity were found to be negatively correlated with changes in miR-1202 expression [54]. The same group reported similar results three years later [55]. In this study, two cohorts of MDD patients (cohort 1 *n* = 55 and cohort 2 *n* = 124) were examined, who differed with respect to how the miRNA expression was examined and how the symptoms of depression were measured. It was found that in both study cohorts, patients who responded to 8-week treatment with escitalopram or desvenlafaxine had lower expression of miR-1202 compared to non-responders. In both study cohorts, responders showed an increase in miR-1202 expression following treatment, while non-responders either showed no change or an increase in the second cohort. Additionally, only in the first cohort was there a correlation between change in expression and change in symptoms. The study found that miR-16 expression differed between responders and non-responders in the first cohort at baseline. Responders, similar to what was observed in the case of miR-1202, had lower expression of miR-16 than non-responders, and this difference persisted even after treatment. In the second cohort, an increase in miR-16 expression was observed among responders following treatment but not among non-responders. Lastly, in contrast to the first cohort, the second cohort showed a pattern similar to miR-1202 for miR-135a expression. Specifically, responders in the second cohort had lower expression of miR-135a at baseline compared to non-responders. Following treatment, an increase in miR-135a expression was observed only among responders, unlike non-responders who showed no change [55]. An increase in miR-135a expression was also found in a separate study that did not differentiate between responders and non-responders. This increase was observed among patients who underwent 16 sessions of CBT but not among patients treated with escitalopram for 12 weeks. It is important to note that patients undergoing CBT were also taking medication. Furthermore, no significant differences were observed in the expression of miR-16 between the two groups or before and after the treatment [56]. According to research by Kim et al. (2019a), miRNA-16-5p levels in the blood at baseline can predict which individuals diagnosed with depression will be cured after receiving duloxetine for 6–8 weeks in two out of three trials [57]. Additionally, the miRNAs miR-21-5p, miR-30b-5p, and miR-146a-5p, which have been found to be important in other studies [57,58,59], were also found to be predictors of duloxetine efficacy in two out of three trials. Furthermore, miR-23a-3p was found to be a predictor of duloxetine efficacy in all three trials and also in the placebo trial as a discriminator between responders and non-responders from baseline blood [57]. However, other studies have yielded inconsistent results. For example, while Kim’s study found that miR-164a had effective predictive capabilities for antidepressants, two other studies did not find this but did identify that it is low among those suffering from depression and increases after antidepressant treatment. However, no difference was found between responders and non-responders in baseline levels of miR-164a [58,59]. These inconsistencies may be due to differences between the studies, as Kim only examined the effect of duloxetine, while the other two included several classes of antidepressants. Reviewed papers are summarize in Table S2.

### 3.3. Other Disorders

#### 3.3.1. Borderline Personality Disorder (BPD)

A study by Perroud et al. (2013) found that individuals with borderline personality disorder (BPD) had significantly higher levels of methylation in specific regions (UTRs I and IV) of the *BDNF* promoter compared to a control group. Interestingly, the study also found that after undergoing psychotherapeutic treatment (dialectical behavior therapy), non-responders to the treatment had a further increase in *BDNF* methylation, while responders to the treatment showed a decrease in methylation [60]. A separate study by Thomas et al. (2018a) did not find similar results to the study by Perroud et al. (2013) when analyzing blood samples from BPD patients and healthy controls. However, the study did find that BPD patients had significantly higher DNA methylation at two specific sites in the *BDNF* promoter at UTR IV in saliva samples compared to healthy controls. However, this increase in methylation did not correspond to a reduction in symptoms in individual patients. Furthermore, Thomas et al. (2018a) also found no correlation between DNA methylation at *BDNF* promoter IV in blood and saliva samples [61]. This discrepancy highlights the importance of selecting a specific tissue for measurement, which will be discussed in more detail later. Additionally, the difference in results could be due to the variation in the methodologies used to analyze methylation in the two studies, with the first study focusing on the average methylation of *BDNF* and the second study focusing on specific CpG sites. One more study concerning BPD tried to use epigenetics to predict therapy efficiency. It showed that DNA methylation levels of *APBA3* and *MCF2* genes before treatment were significantly higher in BPD that responded well to therapy than in those with no symptom improvement [62].

#### 3.3.2. Obsessive–Compulsive Disorder (OCD)

Recent studies have investigated the potential of DNA methylation to predict the efficacy of OCD treatment [63,64,65]. In 2020, Schiele et al. examined the association between methylation of the *MAOA* promoter and the efficacy of CBT therapy in 14 OCD patients. They discovered that individuals with OCD at baseline had lower methylation levels than healthy participants and that the change in methylation levels between baseline and after treatment was negatively associated with improvement in symptoms among those with OCD [63]. In 2021, Schiele et al. observed that moderate reduction in OCD symptoms was associated with average baseline *SLC6A4* methylation levels and with methylation levels at specific CpG sites [64]. Lastly, Schiele et al. showed that methylation of *OXTR*, 10 weeks before OCD-specific cognitive behavioral psychotherapy, was classified and distinguished between responders and non-responders to six weeks of antidepressants. Responders had lower methylation at baseline compared to non-responders, with a negative correlation between methylation at baseline, and symptom severity was demonstrated. These findings particularly concerned obsession symptoms [65]. Similar results were later replicated with a larger sample size [66].

#### 3.3.3. Attention-Deficit/Hyperactivity Disorder (ADHD)

A recent study has shown that there may be a link between epigenetics and the response to treatment for ADHD in children. The study, which included 111 children diagnosed with ADHD, found that those treated with methylphenidate (MPH) for 6 weeks had different methylation levels at the *DAT1* (also known as *SLC6A3*) promoter. When comparing children who responded well to treatment with those who did not, the researchers found that the mean *DAT1* methylation levels did not vary significantly between the two groups. However, they observed that a reduction in methylation levels was strongly associated with an improvement in ADHD symptoms [67]. In line with these findings, a study found that methylation at two specific CpG sites in the *DAT1* 5′-UTR sequence (not the promoter) at the beginning of treatment was positively correlated with a change in the severity of social and psychiatric functioning between pre- and post-treatment among ADHD children who underwent 6 weeks of psychotherapy and/or MPH treatment. However, this correlation was only observed in patients who carried at least one 9-repeat allele and not in patients who were homozygous for the 10-repeat allele [68]. Ding et al. also examined the *DRD4* gene, but no associations were detected [67]. Later, Wang et al. showed that miR-140-3p, miR-27a-3p, miR-486-5p, and miR-151-5p revealed distinct patterns of expression between responders and non-responders among the 92 ADHD patients who completed the 12 months of MPH treatment [69].

#### 3.3.4. Alcohol Use Disorder (AUD)

A study conducted on 93 alcohol dependence (AUD) patients who were treated with naltrexone (NTX), an antagonist to opioid receptors known to reduce alcohol’s reinforcing effect [70], and which examined the influence of *OPRM1* promoter methylation on AUD relapse after NTX therapy did not find any effect of methylation [71]. In line with Lin’s results, Schacht et al. (2022) also did not find *OPRM1* methylation to be an independent predictor of NTX response among AUD patients but did find that NTX effectiveness was moderated by an interaction between methylation of *OPRM1* and *SLC6A3* and *COMT*. AUD patients, who had baseline lower methylation levels of the *OPRM1*, *SLC6A3*, and *COMT* promoters, or the *SLC6A3* (3’UTR), had fewer days of heavy drinking compared to those who had higher *OPRM1* and *SLC6A3* or *COMT* promoter methylation after 16 weeks of NTX therapy [72].

#### 3.3.5. Anorexia

A recent study involving 129 anorexic women who received outpatient psychotherapy treatment for ten months found that methylation levels of the leptin (*LEP*) gene in blood samples collected before treatment can accurately predict treatment outcomes. The study found that women who had the best response to treatment (as measured by changes in body mass index (BMI) and scores on the Structured Interview for Anorexic and Bulimic Disorders) had lower levels of methylation compared to those who had a partial response or no response to treatment. Additionally, the study observed significant changes in methylation levels before and after treatment among women who fully recovered from their eating disorders, and there was also a tendency towards significance among women who had a partial recovery. To validate these findings, the study calculated a cut-off value of 31.25% methylation based on a cumulative distribution analysis evaluated on a replication sample of 33 women. The study found that patients with *LEP* DNA methylation levels below the cut-off demonstrated an increase in BMI over time and during treatment, while patients with *LEP* DNA methylation levels above the cut-off showed a reduction in BMI over time (non-response) [73]. Reviewed papers of other disorders are summarized in Table S3.

In conclusion, while differential methylations in several genes and miRNAs have been replicated in multiple studies, such as *NR3C1* and *FKBP51* in PTSD and miR-1202, miR-146a-5p, and *SLC2A4* in treatments for MDD, among others, further replication is needed to establish accurate data that can be used as a predictive tool for treatment efficacy. It is important to note that the type of treatment, whether pharmacological or psychotherapeutic, how individuals were diagnosed before and after treatment, and the biological markers and how they were examined are important classifications across research studies. The following section will discuss these topics and others in more detail.

## 4. Challenges and Questions for Prediction Studies

**Figure 2:**
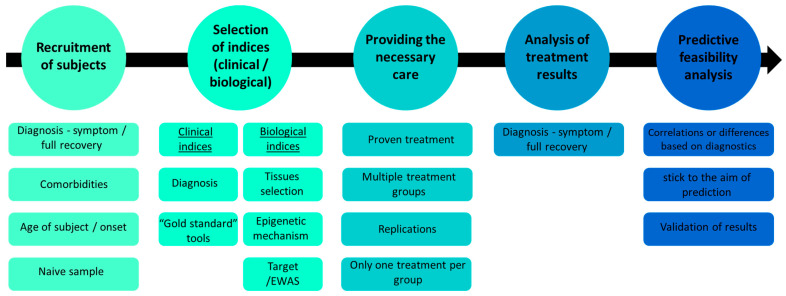
Overview of the proposed experimental design for epigenetic personalized medicine drug discovery, highlighting key stages and considerations.

A prediction study is a well-planned research endeavor that takes into account various aspects of the experimental process. In the following section, we will discuss the concerns and challenges associated with each research design step, outlined in Figure 2.

### 4.1. Recruitment of Subjects

Typically, the selection of the investigated population is based on the disorder of interest, and the enrolled participants are from a recognized health provider that provides the treatment for the disorder(s). When conducting a study on the effectiveness of treatment for psychiatric disorders, it is essential first to clarify the objectives of the chosen treatment, such as symptom relief or complete recovery/remission. This aspect will also be discussed in further detail in subsequent sections. From this perspective, various treatments, such as SSRIs, may be suitable for various disorders [74]. Consequently, it is possible to view these treatments as tools to diminish common symptoms but not necessarily specific disorders. Some studies that recruited participants with various disorders and analyzed biomarkers for treatment efficacy were not always successful (e.g., [75]). Therefore, careful consideration must be given to the choice of study cohort. If the goal is to predict complete recovery from the disorder rather than symptom relief, it is crucial to determine whether the participants have comorbidities, as mental disorders often coexist with other disorders [76]. To isolate the analyzed disorder, it is important to select a group with similar comorbidities or a single disorder. Additionally, it is worth noting that different disorders can manifest at different ages [77], which can be influenced by various factors, with the environment playing a major role.

Epigenetics, which examines the relationship between the environment and gene expression, may hold the key to understanding the diversity of disorder onset. The field of epigenetics has been extensively studied in relation to aging and is considered one of the hallmarks of aging [78]. Therefore, subjects with different disorders may be influenced by their age and the sensitivity of their epigenetic profile to this characteristic. It is important to note that this does not mean that all subjects need to be the same age, as an illness may manifest at various ages, but if the disorder being investigated is known to manifest around a specific age (e.g., eating disorders [79]), this should be taken into account in the selection of the study cohort.

Lastly, most of the studies reviewed here examined a cohort that had received prior treatments. Quite a few studies have examined the efficacy of a particular treatment while additional treatments occur in the background during the study; therefore, it is of the utmost importance to isolate the effect of the treatment being studied. One of the benefits of using epigenetics in studies predicting the effectiveness of multiple treatments is that the epigenetic profile can change in response to environmental conditions, including past or current drug use. This can provide valuable insights into the potential impact of different treatments on gene expression and disease progression. Drug exposure throughout fetal development may program interindividual variances in hepatic drug metabolism ability. Exposure to medicines or drug receptor ligands during fetal life caused epigenetic alterations of specific genes that altered absorption, distribution, metabolism, and excretion (ADME) gene expression in adult mice, an epigenetic imprinting example of drug metabolism [5]. When it comes to drug metabolism in adult humans, an epigenetic component may be influenced by exposure to certain medications and other xenobiotics when in utero or during early infancy [80].

If the ultimate goal of personalized medicine is to tailor treatment according to the patient’s profile, taking into account their health history, then the impact of previous drug use on the patient should not impede the achievement of this goal. However, studying patients who have not previously been treated with medication can provide valuable insight into a patient’s initial and final therapy and thus provide additional value to the research. This presents a challenge from a procedural perspective, as recruiting subjects based on their initial treatment-seeking behavior can be difficult, and it requires careful selection and screening to ensure that subjects have not previously been treated for the disorder in question. However, there are databases, such as PSYcop [81], that allow for the use of preliminary data on individuals, and it is expected that these databases will expand as the field develops. The more datasets are shared, the more accurate the results will be, and the replication capacity will be increased. This will lead to clinically relevant findings and potentially result in clinical clearance.

### 4.2. The Selection of Parameters (Clinical and Biological)

The measurements used in the study, both for diagnosis and predictive analysis, are crucial for various reasons, including the ability to replicate the study, compare it to other studies, and verify previous findings. Consistency in measurement methods is essential to ensure that the results are reliable and valid, and it also enables cross-study comparisons, which can help understand the findings’ generalizability. Therefore, the choice of measurement tools should be made carefully and based on established and validated methods.

#### 4.2.1. Clinical Parameters

Diagnosing subjects for a study can be a complex task, particularly when it comes to psychiatric disorders. The standard method, based on diagnostic manuals, such as the DSM and ICD, is widely used but has been criticized for its limitations in capturing the complexity and individuality of patients. Alternative nosological approaches, such as categorical, dimensional, and hybrid methods, have been proposed to address these limitations [82]. However, regardless of the approach used, it is important to clearly define the research objectives, whether it be predicting improvement in symptoms or complete recovery from the disorder. It should also be acknowledged that recovery from psychiatric disorders is often not complete, and that sound therapy can alleviate symptoms and improve quality of life, but that most mental health problems are not curable in the same way as many medical conditions. The challenges and limitations of psychiatric nosology are discussed in more detail in [82].

In addition, there are many assessment tools for symptoms in different disorders. In order to compare and replicate results, it is crucial to use the “gold standard” in each field. For example, most of the studies reviewed here regarding MDD used the Hamilton Depression Rating Scale (HAM-D) to assess MDD symptoms. Therefore, it is vital to use established and validated assessment tools for each disorder. It is important to note that this review does not aim to evaluate or rank specific assessment tools but rather to highlight the complexity of the assessment process.

#### 4.2.2. Biological Parameters

To a considerable extent, the human DNA methylome is variable due to the wide variety of human tissues and the fact that methylation is tissue-specific. One significant challenge in the field of epigenetics research is the difficulty in obtaining human brain samples, which often necessitates the use of substitute tissues. This issue is particularly prevalent in the study of psychiatric disorders, as the brain plays a crucial role in the development and manifestation of these conditions. As a result, researchers must carefully consider the suitability of substitute tissue samples and the potential limitations of using them in their studies. Despite these challenges, there has been progress in the development of alternative sample types, such as blood, saliva, and buccal swabs, for the analysis of methylations or miRNAs to predict the efficacy of therapy for mental illnesses [35,36,61,68]. Determining the most appropriate tissue for analysis of methylations or miRNAs to predict the efficacy of therapy for mental illnesses is a complex task. Sample collection should be simple, inexpensive, and as non-invasive as possible and fulfill the ultimate objective of individualized therapy. While blood is a commonly used tissue due to its ability to mirror biological function, it also has several drawbacks. Decentralized investigations may face challenges in collecting blood samples due to a lack of trained phlebotomists and logistical support. Moreover, blood drawing can be painful for sensitive populations, such as children, and can be avoided. In a recent review, saliva and blood were compared as tissue samples for epigenetic biomarker studies, with a preference for saliva, specifically for DNA methylation [83].

Recent studies have shown that findings obtained from blood samples can be replicated in saliva samples [84] However, other studies have also identified blood-specific anomalies that were not replicated in saliva samples [61]. As explained by Thomas et al., it is essential to examine the tissue-specificity of DNA methylation in biomarker investigations, as different cell types may contribute to variations in methylation levels. For example, their study investigated *BDNF* methylation in exfoliated epithelial cells and lymphocytes, which travel from the bloodstream to the oral cavity. BDNF is known to be expressed by both cell types, and there is evidence of lymphocyte enrichment in oral samples, compared to blood, which may result in differential epigenetic control of this cell type being more visible in saliva than in blood [61]. This explanation effectively demonstrates one method for selecting tissue for methylation tests. However, it is also important to mention a perspective that may be less convenient for optimists; there is a viewpoint that suggests that mental illness is solely a brain disease and that other tissues are not relevant for epigenetic analysis due to the significant variation in epigenetics between different tissues. Cariaga-Martinez and Valo-Paz have proposed that the epigenetic approach may not be appropriate for studying mental illness if testing is conducted on tissues other than the brain. They argue that using other tissues may lead to confusing and unproductive results, even if they seem promising [85]. Yet, recent studies have shown that DNA methylation levels at several loci do correlate between peripheral tissues and the brain [86,87], which challenges this viewpoint. Another answer to the claim of Cariaga-Martinez and Valo-Paz will be given in the last part of this review. To summarize, it is critical to thoroughly research the literature to determine whether there is a more appropriate tissue for the gene or site of interest in question. This can enhance the study’s outcome by allowing for more accurate replication and the opportunity to conduct additional studies in different tissues.

Another important consideration in research that aims to predict treatment efficacy through epigenetic mechanisms is the choice of mechanism to study. The majority of studies focus on either DNA methylation or miRNA. For both, researchers must decide whether to study a specific target or conduct a screening, such as using illuminaEPIC for DNA or RNAseq for RNA. While each technique presents its own challenges and limitations, note that this is a common issue in epigenetic research and one which will not be extensively discussed in this review.

In certain studies, evidence has been presented for the downstream biological consequences of methylation (for example, [51]); although these findings can greatly contribute to the etiology and understanding of a certain disorder, they are not required in epigenetics prediction studies per se.

### 4.3. Providing the Necessary Care

There are several crucial elements of an administered treatment that can be predicted. Firstly, it is essential to ensure that the therapy chosen has been proven to be effective in treating the disorder in question, whether through symptom reduction or complete recovery. Additionally, the duration of treatment must be supported by evidence. For instance, a noticeable response to antidepressant medication requires an average of four weeks, whereas symptom remission in MDD requires six weeks [88].

Second, the primary objective is to forecast the most effective treatment for the patient; thus, it is critical to examine and compare multiple treatment options within the same study, such as SNRI and SSRI treatment, both of which are known to aid those with depression and anxiety disorders. However, only a single study reviewed in this report examined and distinguished between the two types of antidepressant medications [43]. Such trials will not only determine who will benefit from the treatment, but also which treatment will be most effective.

As previously mentioned, treatment choice is a crucial factor to consider. In order to accurately evaluate the effectiveness of a treatment, each subject must receive only one treatment, not multiple treatments simultaneously. Additionally, participants should be naïve, meaning that they have not previously received treatment for the illness being examined. When treatment is administered alongside other treatments, it can only provide limited information that must be interpreted with caution. In addition, early exposure to drugs can also affect the epigenetic profile [89]. Furthermore, significant added value can be gained by replicating the findings of earlier studies that used similar treatment approaches.

### 4.4. Analysis of Treatment Results

The central concern in this section centers around the diagnostic method for the subject and determining whether the clinical indicator is based on symptoms or complete recovery, as discussed above.

### 4.5. Predictive Feasibility Analysis

When conducting studies to predict a drug treatment’s efficacy, it is important to consider the approach used to measure the biological markers that will be used to predict the response to treatment. One approach is to use a dichotomous separation, where the biological markers are used to distinguish responders from non-responders, that is, to identify differences between those who respond to treatment and those who do not. Another approach is to use a more flexible method, such as a cut-off point, to determine a correlation between the biological parameters and the severity of symptoms before treatment or the change in symptoms after treatment. In either scenario, we can obtain predictive information regarding the success of the treatment based on the biological parameters obtained before the therapy. In this context, two points should be made: first, many studies show that there is or is not a change in the biological parameters before and after the treatment, and that there is or is not a relationship between the change in the biological parameters and a change in the symptoms. While these studies contribute significantly to the etiology of the disorder, the information they provide does not help to predict the outcome of the condition. Second, in contrast to the perspective presented by Cariaga-Martinez and Valo-Paz, according to which mental disorders originate solely in the brain, it has been demonstrated that it is possible to predict the effectiveness of treatment using measures taken from peripheral tissue, such as blood. This suggests that the origin of a disorder may not be as crucial in determining treatment effectiveness. It should be noted that understanding the mechanism of the disorder is still of importance but that it is not necessarily required for accurate prediction (e.g., [49]).

Lastly, it is important to note that there are statistical challenges to consider when analyzing epigenetic information, such as determining whether a specific or unbiased approach, such as EWAS, should be used, and validating results through downstream methods, such as RNA expression or other previously established techniques [90,91].

### 4.6. Machine Learning and Artificial Intelligence

In the rapidly evolving field of epigenetics, machine learning and artificial intelligence are promising for advancing our understanding of mental disorders and predicting drug efficacy [91,92]. Machine learning techniques can be utilized to identify epigenetic biomarkers and predict disease outcomes. Network-based machine learning approaches, such as deep learning and graph-based algorithms, can integrate epigenetic data with other omics data to improve treatment efficacy predictions [93,94]. Furthermore, the complex interplay between genetic and environmental factors in mental disorders can be better understood by investigating the relationships between genetic variations, epigenetic modifications, and treatment outcomes. By leveraging machine learning and artificial intelligence approaches, epigenetics has the potential to revolutionize the understanding and treatment of mental disorders by identifying biomarkers, elucidating complex gene–environment interactions, and discovering novel therapeutic targets.

### 4.7. Safety Issues

The use of epigenetic biomarkers for predicting treatment efficacy in psychiatric disorders has the potential to revolutionize personalized medicine approaches. Nevertheless, it also poses significant safety concerns that cannot be overlooked. Improper treatment of psychiatric disorders based on incorrect predictions of treatment efficacy using epigenetic profiles can lead to significant negative consequences for patients, their families, and society, while undermining the overall effectiveness of personalized medicine approaches. False positives occur when an epigenetic biomarker inaccurately indicates that a particular treatment will be effective for a patient. This can lead to inappropriate or ineffective treatment that may exacerbate psychiatric symptoms, leading to increased distress and functional impairment in patients [95]. In contrast, false negatives occur when an epigenetic biomarker incorrectly suggests that a treatment will not be effective for a patient. This can lead to untreated or improperly treated psychiatric disorders, increasing the risk of suicide or suicidal ideation [96].

Mistreatment may result in prolonged periods of distress, reducing patients’ quality of life and potentially causing long-lasting emotional, social, and occupational difficulties. This can place emotional and financial burdens on patients’ families, leading to stress, conflict, and strained relationships [97]. The mistreatment of psychiatric disorders can also contribute to homelessness, unemployment, and involvement with the criminal justice system [98]. Finally, it is essential to note that mistreatment and lack of recovery may perpetuate the mental illnesses stigma, further alienating patients from their communities and support networks [99].

Researchers should adopt several strategies to address the safety issues associated with using epigenetic biomarkers for predicting treatment efficacy in psychiatric disorders. First, studies should have rigorous study designs, including large sample sizes, appropriate controls, and replication cohorts, to increase the reliability of identified epigenetic biomarkers. Second, integrative approaches that combine epigenetic information with other types of biomarkers (e.g., genetic, proteomic, and neuroimaging data) should be used to improve the accuracy of treatment efficacy predictions. Third, researchers should develop and validate more precise and reliable epigenetic profiling methods to minimize the risk of false positives and negatives. Fourth, regular assessment and refinement of research methodologies and treatments based on evolving knowledge of epigenetics and its role in psychiatric disorders is essential. Fourth, ethical considerations must be addressed by ensuring transparency, obtaining informed consent, and safeguarding patient data. Finally, the implementation of machine learning and artificial intelligence can considerably minimize the risks associated with prediction errors and increase the accuracy of forecasts. These strategies can minimize risks and improve the development and application of epigenetic biomarkers for personalized medicine in mental disorders. In addition, a comprehensive review by Santalo and Berdasco (2022) explains the main challenges in epigenetic research in the technical, ethical, and social dimensions [100]. It is also worth noting that some psychiatric drugs can affect epigenetic mechanisms, further underscoring the need to be cautious about mistreatment [89].

## 5. Conclusions

The field of mental health treatment is constantly evolving, and one area that has garnered significant attention in recent years is the potential use of epigenetics as a predictor of treatment efficacy. In this review paper, we aimed to provide a comprehensive overview of the current state of research in this field.

First, we emphasized the importance of forecasting treatment efficacy in mental health. With a growing number of individuals seeking help for mental health issues, it is crucial that we have the tools and understanding to provide effective treatment. However, the treatment process is not always straightforward and can often be a trial-and-error process. By using epigenetics as a predictor, we may understand better which treatments may be more effective for specific individuals. We then reviewed the existing studies that have attempted to use epigenetics as a predictor of treatment efficacy. While the field is still in its infancy, a few studies have shown promising results. For instance, some studies have found that epigenetic markers, such as DNA methylation, can predict treatment response in major depressive disorder. However, the literature on the subject is still limited, and further research is needed to confirm these findings. Additionally, we discussed the challenges and limitations of using epigenetics as a predictor of treatment efficacy. For example, epigenetic changes can be influenced by a wide range of environmental and lifestyle factors, making it difficult to establish causality between epigenetic markers and treatment response. Moreover, the complexity of the human genome and the epigenome presents a challenge for identifying specific biomarkers that can predict treatment response. Finally, we proposed future directions for research in this field. We suggested that future studies should aim to replicate and validate the findings of previous studies and explore the potential use of epigenetics as a predictor of treatment efficacy in other mental disorders. Additionally, it would be valuable to investigate the feasibility of using epigenetics as a predictor in clinical settings.

Some limitations should be acknowledged in the current review. Firstly, the search strategy was not systematic, and therefore some relevant articles may have been missed. Although efforts were made to be as inclusive as possible, we cannot rule out the possibility of some bias in the selection of studies. Secondly, while this review focused on two key epigenetic mechanisms, namely, DNA methylation and non-coding RNA, there are other epigenetic modifications that were not extensively discussed. While it is unlikely that these mechanisms significantly affect the conclusions drawn in this study, future research should aim to address their potential contributions to the broader epigenetic landscape.

In conclusion, the potential use of epigenetics as a predictor of treatment efficacy in mental health is a promising area of research that should be taken seriously. The current literature suggests that epigenetics may play a significant role in treatment response, and investing in further research in this field is essential. The potential benefits of utilizing epigenetics to improve our understanding of treatment efficacy in mental disorders is a valuable pursuit that should not be overlooked.

## Supplementary Materials

The following supporting information can be downloaded at: https://www.mdpi.com/article/10.3390/cells12081173/s1, Table S1: Summarized papers on anxiety disorders, panic disorders, phobias, and PTSD; Table S2: Summarized papers on MDD; Table S3: Summarized papers on different disorders reviewed in the “Other Disorders” section.
