# Supplementary material for: Can Epigenetics Predict Drug Efficiency in Mental Disorders?
Source: Cells. 2023 Apr 17;12(8):1173. doi: 10.3390/cells12081173 (PMC10136455; doi:10.3390/cells12081173)
Supplement: Supplementary file 1 [file cells-12-01173-s001.zip › cells-2222511-supplementary.pdf]

Table S1. Summarized papers of Anxiety disorders, panic disorders, phobia, and PTSD.

Paper	Psychiatric disorder	Tissue	Epigenetics mechanism	Psychiatric measurements	Groups and treatment	Findings			information
						Genes	Methylation /expression Status	Disorder/ symptom status	
(Yehuda et al., 2013)	PTSD – 16 combat veterans	Blood – PBMCs	bisulfite sequenced	SCID CAPS PSS-SR CTQ DRRI	12 weeks of PE Responders (n=8) Non-responders (n=8) Determine by presence or absence of PTSD based on CAPS	*NR3C1*	Pre	Responders >Non-responders	prediction
						FKBP51	pre > post	Responders	Track symptom change
							pre < post	Non-responders	
(Pape et al., 2018)	PTSD - 88 female patient	whole blood	Illumina 450K DNA methylation arrays	CAPS CTQ PSS-SR	6 weeks of treatment of CRF1 receptor antagonist (GSK561679).	*NR3C1*	⬆pre	⬆△PTSD symptoms (among abused-as-children-women)	prediction
						FKBP51	None		
(Bishop et al., 2018)	PTSD – 22 combat veterans	whole blood	bisulfite sequenced (Illumina MiSeq)	CAPS PCL	9 weeks of MBSR Responders (n=11) Non-responders (n=11) Determine by reduction of 10 or more points on PCL while The minimal clinically important difference (MCID) for PTSD	*SLC6A4*	None		
						FKBP51	pre > post	Responders	

					symptom severity is a reduction of 10 or more points on the PCL and CAPS.		pre < post	Non-responders	Track symptom change
(Yang et al., 2021)	PTSD – 97 combat veterans	whole blood	Infinium Human-Methylation450 BeadChip	CAPS SCID	PE therapy<br>Responders (n=20)<br>Non-responders (n=22)<br>Determine by presence or absence of PTSD based on CAPS	*NR3C1*	Pre	Responders <Non-responders (barely significant)	prediction
							⬇methylation pre to post	⬆△PTSD symptoms improvement (only among responders)	Track symptom change
						FKBP51	pre > post	Responders	Track symptom change
							pre = post	Non-responders	
						PEX5	Pre	Responders >Non-responders	prediction
							⬆△methylation	⬆△PTSD symptoms improvement	Track symptom change

						ALOX15B	Pre	Responders >Non-responders	prediction
							⬆△methylation	⬆△PTSD symptoms improvement	Track symptom change
						SDK1	Pre	Responders >Non-responders	prediction
							pre > post	Responders	Track symptom change
							pre < post	Non-responders	Track symptom change
(Roberts et al., 2019)	AG/AG&PD/SsP (n=111)	Whole blood	Sequenom EpiTyper	DIPS CGI-S	CBT (Duration of treatment varies)<br>Treatment response was defined as change in CGI-S score	*FKBP51*	⬇methylation pre to post	⬆△PTSD symptoms improvement	Track symptom change
(Roberts et al., 2015)	AD – 98 childrens	buccal swabs	Sequenom EpiTYPER	ADIS-IV-C/P (or German equivalent (Kinder-DIPs)	CBT (Duration of treatment varies),<br>Treatment response was defined as change in primary anxiety disorder severity.<br>Remission was regarded as the	*FKBP51*	pre	None	
							⬇methylation pre to post	⬆△PTSD symptoms improvement (only among	Track symptom change

				CSRs	absence of the primary anxiety according to diagnostic criteria			"risk" allele carriers)	
(Roberts et al., 2014)	AD – 116 childrens	buccal swabs	Sequenom EpiTYPER	ADIS-IV-C/P	CBT (Duration of treatment varies)<br>Responders and Non-responders<br>Determine by presence or absence of the primary anxiety diagnosis	*SERT*	Pre < post	Responders	Track symptom change
							pre > post	Non-responders	Track symptom change
(Ziegler et al., 2016)	PD – discovery (n=28),	Whole blood	bisulfite sequenced	SCID-I	6-week CBT,<br>Patients showing a decrease in the panic attacks defined responders increased or no change Non-responders (discovery sample, Res = 11, Nres= 17) (Replicated sample, Res = 8, Nres= 8)	*MAOA*	Pre	Responders ≠ Non-responders	prediction
							⬆Pre methylation	⬇Pre PD severity	Track symptom
							pre < post	Responders	Track symptom change
							pre > post	Non-responders	Track symptom change
	PD+AG-replicated (n=16)			CAPI-WHO-CIDI			⬆Pre methylation	⬇Pre AG severity	Track symptom
(Ziegler et al., 2019)	PD (N=57)	Whole blood	Illumina MethylationEPIC BeadChip (EWAS)	SCID-I	6-week CBT,<br>at least 50% reduction in HAM-A defined as responders (n=20)	*IL1R1*	pre < post	Responders	Track symptom

					, all other non-responders (n=27)		pre = post	Non-responders	
(Min et al., 2019)	PD (N=60)	Blood - plasma	Human panel I+II V4.M (Exiqon) RT-PCR	PDSS HAMA14 HAMD17	6 weeks sertraline, clinical respones - 40% reduction in pdss score from base line (n=32) 2. clinical remmision, HAMA14<7 (n= 28)	miR-25-3p	⬆△expression	⬆△PDSS3 (the severity of expected anxiety) ⬆△PDSS7 (the impairment of social function)	Track symptom
						miR-660-5p	⬆△expression	⬆△HAMA5 (memory and attention disorders) ⬆△PDSS7 (the impairment of social function)	Track symptom
						△miR-148-5p	⬆△expression	⬆△ PDSS4 (scene scared and/or avoidance) ⬇△HAMA1	Track symptom

								3 (autonomic nerve symptoms)	
						△miR-144-5p	⬆△expression	⬇△HAMA9 (symptoms of cardiovascular system)	Track symptom

Table S2. Summarized papers of MDD.

Paper	Psychiatric disorder	Tissue	Epigenetics mechanism	Psychiatric measurements	Groups and treatment	Findings			information
						Genes	Methylation Status	Disorder/symptom status	
(Powell et al., 2013)	MDD (n=113, GENDEP)	Whole blood	Sequenom EpiTYPER	MADRS	12 weeks of escitalopram (*n*=80) or nortriptyline (*n*=33)	*IL11*	⬇pre (CpG1)	⬆response	prediction
							⬆pre (CpG2)	escitalopram : ⬆response nortriptyline: ⬇ response	prediction
(Okada et al., 2014)	MDD (n=40)	Whole blood	Sequenom EpiTYPER	HAMD	6 weeks of various antidepressants types, betters responders : improvement ratio (IR) of 50% or more poorer responders: patients with an IR of less than 50%	SLC6A4	pre	Better responders > poorer responders	prediction
							⬆pre	⬆pre	prediction
(Domschke et al., 2014)	MDD (n=94)	Whole blood	bisulfite sequenced	SCID HAMD BDI GAF	6 weeks of escitalopram, Clinical remission was defined as subjects having a HAMD total score ≤ 7 after treatment	SLC6A4	⬆pre	⬆response	prediction
(Schiele, Zwanzger, et al., 2021)	MDD (n=236)	Whole blood	bisulfite sequenced	SCID HAMD	6 weeks with SSRI or SNRI, at least 50% reduction in HAMD defined as	SLC6A4	⬆pre	⬆response	prediction

				BDI<br>GAF	responders , all other non-responders.				
(Tadić et al., 2014)	MDD (n=39)	Whole blood	Bisulfite sequencing	HAMD	6 weeks treatment of antidepressant<br>at least 50% reduction in HAMD defined as responders, others as non-responders.	BDNF	pre	Responders>Non-responders	prediction
							pre=post	For all sample	
(Wang et al., 2018)	MDD (n=85)	Whole blood	Bisulfite sequencing	HAMD-17<br>LES<br>CTQ	8 weeks of escitalopram<br>Clinical remission was defined as subjects having a HAMD total score ≤ 7 after treatment. Remmiters (n=40) Non-remmiters (n=45)	BDNF	pre	Remmiters>Non-remmiters	prediction
							⬆pre	⬆response	prediction
							pre<post	Remmiters	Track symptom
							pre=post	Non-remmiters	
(Ju et al., 2019)	MDD – discovery (n=177),	whole blood	Illumina MethylationEPIC BeadChip and bisulfite sequencing	MADRS	8 weeks of escitalopram, responders (n=82) - ≥50% decrease in MADRS after treatment, non-responders (n=95)	CHN2	Pre (cg23687322)	Responders<Non-responders	prediction
							Pre (cg06686818)	Responders<Non-responders	prediction
						JAK2 (cg08339825)	Pre	Responders<Non-responders	prediction
	MDD - replicated (n=147)	whole blood	Illumina Methylatio	HAMD	8 weeks of escitalopram, responders (n=71) - ≥50% decrease in HAMD after	CHN2 (cg06686818)	pre	Responders<Non-responders	prediction

			nEPIC BeadChip		treatment, non-responders (n=76)				
(Domschke et al., 2015)	MDD (n=94)	Whole blood	bisulfite sequenced	SCID<br>HAMD<br>BDI<br>GAF	6 weeks of escitalopram, Clinical remission was defined as subjects having a HAMD total score ≤ 7 after treatment	MAO-A	no finding associated with symptom improvement or remmission		
(Kahl et al., 2016)	MDD (n=37)	Whole blood	bisulfite sequencing	MADRS<br>SCID	6 weeks of CBT, remission, assessed as a MADRS sum score < 10	glut1<br>glut4			
(Lopez et al., 2014)	MDD (n=32)	Whole blood	Human miRNA microarray	HAMD	8-weeks of citalopram, remmiters (n=16) non-responders (n=16), classified based changes in HAMD	miR-1202	pre	Remmiters<Non-responders	prediction
							Pre<post	Remmiters	Track symptom
							Pre=post	Non-responders	Track symptom
							⬆△expression	⬇%△HAMD	Track symptom
(Fiori et al., 2017)	MDD (Cohort1 n=55)	Whole blood	Taqman assays	HAMD	8 weeks of escitalopram (n=27) or desvenlafaxine (n=28), responders (n=31) - ≥50% decrease in HAMD after treatment, non-responders (n=24)	miR-1202	pre	responders <Non-responders	prediction
							Pre<post	responders	Track symptom
							Pre=post	Non-responders	
							⬆△expression	⬇△HAMD	Track symptom

						miR-16	pre	responders <Non-responders	prediction
							Pre=post	Responders and Non-responders	
						miR-135a	none		
	MDD (Cohort2 n=124)	Whole blood	Firefly BioWorks miRNA multiplex assay	MADRS	8 weeks of duloxetine, responders (n=97) - ⩾50% decrease in MADRS after treatment, non-responders (n=27)	miR-1202	pre	responders <Non-responders	prediction
							Pre<post	responders	Track symptom
							Pre=post	Non-responders	
						miR-16	Pre<post	responders	Track symptom
							Pre=post	Non-responders	
						miR-135a	pre	responders <Non-responders	prediction
							Pre<post	responders	Track symptom
							Pre=post	Non-responders	
	MDD		qRT-PCR				Pre<post	CBT treatments	

(Issler et al., 2014)		Whole blood		HAMD - (HDRS)	12-weeks of CBT (N=11) or escitalopram (N=11)	miR-135a	Pre=post	escitalopram	
						miR-16	none		
(Kim et al., 2019a)	MDD trial A (n=212)	Blood - plasma	MicroRNA sequencing using Ion Proton Sequencer	MADRS	6-8 weeks of duloxetine, remmision defined by MADRS score ⩽ 10	miR-23a-3p,	pre	responders ≠Non-responders (in 2 or more trial)	prediction
	MDD trial B (n=181)					miR-16-5p,			
	MDD trial C (n=230)					miR-146a-5p			
						miR-21-5p			
(Lopez et al., 2017)	MDD - discovery cohort (DRCT) (n=258)	Whole blood	HiSeq2500 Illumina sequencer validated by Firefly BioWorks miRNA multiplex assay and Human HT-12 v4 Expression	MADRS	8 weeks of duloxetine, escitalopram or nortriptyline responders - ⩾50% decrease in MADRS after treatment, non-responders	miR-146a-5p (DRCT, RPCT1, RPCT2)	Pre>post	responders	Track symptom
	MDD- replication cohort 1 (RPCT1)(n=61), GENDEP					miR-146b-5p (DRCT, RPCT1, RPCT2)	Pre>post	responders	Track symptom

	MDD-replication cohort 2 (RPCT2)(n=158), CAN-BIND project		Bead Chip (Illumina) and aqMan RT-PCR microRNA assays			miR -24-3p (DRCT, RPCT1, RPCT2)	Pre>post	responders	Track symptom
						miR-425-3p (DRCT, RPCT1)	Pre>post	responders	Track symptom
						miR-3074-5p (DRCT)	Pre>post	responders	Track symptom
(Hung et al., 2019)	MDD (n=69)	Blood-PBMCs	qRT-PCR	SCID-I HAMD-17	4 weeks of various antidepressant, Remission was defined as total HAMD-17score≤7	let-7e	⬆pre	⬇symptoms	Track symptom
							Pre<post	remitters	Track symptom
						miR-146a	⬆pre	⬇symptoms	Track symptom
						miR-145	Pre<post	remitters	Track symptom
						MiR-223	Pre<post	remitters	Track symptom
						miR-155	⬆pre	⬆symptoms	Track symptom
							Pre<post	remitters	Track symptom

Table S3. Summarized papers of different disorders reviewed in “Other disorders” section.

Paper	Psychiatric disorder	Tissue	Epigenetics mechanism	Psychiatric measurements	Groups and treatment	Findings			information
						Genes	Methylation /expression Status	Disorder/symptom status	
(Perroud et al., 2011)	BPD (n=115)	Whole blood	high-resolution melt profile	SCID<br>DIGS<br>BDI<br>BHS<br>BIS-10<br>CTQ	4 weeks of I-DBT	BDNF	Pre>post	Responders	Track symptom
							Pre<post	Non-responders	Track symptom
(Thomas et al., 2018a)	BPD (n=26)	Whole blood and saliva	Bisulfite sequencing	SCL90R<br>BSL23<br>CTQ	12-weeks of DBT	BDNF	Salivary: pre>post	All subjects (no correlation to symptoms)	
(Knoblich et al., 2018)	BPD (n=44)	Whole blood	Bisulfite sequencing	SCL90R<br>BSL23<br>CTQ	12-weeks of DBT, A patient was defined as responder if the GSI t-score was reduced by more than 5 points post-therapy and additionally a	APBA3	pre	responders >Non-responders	prediction
						MCF2	pre	responders >Non-responders	prediction

					score lower than 2.05 for the BSL23 was reached responders (n=7), non-responders (n=17)				
(Schiele et al., 2020)	OCD (n=14)	Whole blood	Bisulfite sequencing	SCID-I Y-BOCS	8- to 10-week semi-standardized CBT	*MAOA*	⬆△methylation	⬇△symptoms	Track symptom
(Schiele et al., 2021)	OCD (n=113)	Whole blood	Bisulfite sequencing	SCID-I Y-BOCS BDI CTQ	8- to 10-week semi-standardized CBT	*SLC6A4*	⬇pre	⬇ response	prediction
(Schiele et al., 2021)	OCD (n=113)	Whole blood	Sequenom EpiTYPER	SCID-I Y-BOCS	Responders were define if total Y-BOCS ≤12 after treatment	*OXTR*	pre	Responders<non-responders	prediction
							⬇pre	⬆△response	prediction
(Bey et al., 2021)	OCD (n=98)	Whole blood	Illumina MethylationEPIC BeadChip	SCID-I Y-BOCS CTQ LES	CDT treatment Responders: reduction of 35% in Y-BOCS scores after treatment (*n* = 5	*OXTR*	⬇pre	⬆△response	prediction

					) non-responders (n=40)				
(Ding et al., 2016)	ADHD (n=111)	Whole blood	Bisulfite sequencing	CDIS SNAP-IV	6 weeks of MPH, classification of response as follows: responders: reduction >30% in SNAP-IV (n=65), non-responders (n=46)	*DAT1*	⬇methylation pre to post	⬆response	Track symptom
							pre	Responders=non-responders	
						DRD4	none		
(Adriani et al., 2018)	ADHD (n=30)	buccal swabs	Bisulfite sequencing	SCID CGAS CPRS *SNAP-IV* K-SADS/PL CBCL	6 weeks of psychotherapy and/or MPH	*DAT*	⬆pre	⬆△symptoms (only among carrying at least one 9-repeat allele)	prediction
(Wang et al., 2022)	ADHD (n=92)	White blood cells	qRT-PCR	ADHD-RS WISC-IV	12 months of MPH, Response to therapy was defined as a 30% improvement in	miR-140-3p	Pre>post	responders	Track symptom
							⬆△expression	⬆△ ADHD-RS	Track symptom
							Pre>post	responders	Track symptom

					symptoms compared to the ADHD-RS scores at baseline. In addition, the total ADHD-RS scores ≤ 18. Remitters (n=50), non-remitters (n=42)	miR-27a-3p	⬆△expression	⬆△ ADHD-RS	Track symptom
						miR-30e-5p	Pre>post	responders	Track symptom
						Let-7g-5p	Pre>post	responders	Track symptom
						miR-486-5p	Pre>post	responders	Track symptom
						miR-486-5p	⬆△expression	⬆△ ADHD-RS	Track symptom
						miR-151-3P	Pre>post	responders	Track symptom
						miR-151-5p	Pre>post	responders	Track symptom
						miR-151-5p	⬆△expression	⬆△ ADHD-RS	Track symptom
						miR-126-5p	Pre>post	responders	Track symptom
						miR-150-5P	Pre<post	Non-responders	Track symptom

(Lin et al., 2020)	AUD (n=93)	Whole blood	Sequenom EpiTYPER	SCID	NTX treatment	OPRM1	none		
(Schacht et al., 2021)	AUD	blood mononuclear cells	Illumina MethylationEPIC BeadChip	SCID	16-week treatment of NTX	OPRM1	↓pre (only together with lower methylation of SLC6A3 or COMT)	↓relapse	prediction
(Neyazi et al., 2019)	Anorexia discovery (n=93)	Whole blood	Bisulfite sequencing	PSR EDI-2 PHQ SIAB-Ex	40 weeks of psychotherapy full recovery was defined as a PSR score of 1 or 2 and BMI >18.5	LEP	pre	Responders<non-responders	prediction
							Pre<post	responders	prediction
						LEPR	none		
	Replicated (n=33)					LEP	pre	Responders<non-responders	prediction
